# Relationships between compulsive exercise, quality of life, psychological distress and motivation to change in adults with anorexia nervosa

**DOI:** 10.1186/s40337-018-0188-0

**Published:** 2018-02-05

**Authors:** Sarah Young, Stephen Touyz, Caroline Meyer, Jon Arcelus, Paul Rhodes, Sloane Madden, Kathleen Pike, Evelyn Attia, Ross D. Crosby, Phillipa Hay

**Affiliations:** 1 Griffith Taylor Building, School of Psychology, University of Sydney, Sydney, Australia; 2 School of Psychology, University of Sydney, Sydney, Australia; 3 WMG, University of Warwick, United Kingdom & University Hospitals Coventry and Warwickshire NHS Trust, Coventry, UK; 4 Institute of Mental Health, Faculty of Medicine & Health Sciences, University of Nottingham, Nottingham, UK; 5 Leicestershire Adult Eating Disorders Service, Leicestershire Partnership NHS Trust, Bennion Centre, Glenfield Hospital, Leicester, UK; 6 School of Medicine, University of Sydney, Sydney, Australia; 7 Eating Disorders Service at the Sydney Children’s Hospitals Network, Westmead, Australia; 8 Division of Behavioral Health Services and Policy Research, Columbia University, New York, USA; 9 Department of Psychiatry, College of Physicians and Surgeons of Columbia University, Weill Cornell Medical College, New York, USA; 10 Neuropsychiatric Research Institute, Fargo, North Dakota USA; 11 University of North Dakota School of Medicine and Health Sciences, Fargo, North Dakota USA; 12 Translational Health Research Institute (THRI), School of Medicine, Western Sydney University, Campbelltown, Australia

**Keywords:** Compulsive exercise, Anorexia nervosa, Quality of life, Obsessive-compulsive, Anxiety, Depression, Motivation to change

## Abstract

**Background:**

For people with anorexia nervosa (AN), compulsive exercise is characterized by extreme concerns about the perceived negative consequences of stopping/reducing exercise, dysregulation of affect, and inflexible exercise routines. It is associated with increased eating disorder psychopathology and poor clinical outcome. However, its relationships with two important clinical issues, quality of life (QoL) and motivation to change, are currently unknown. This study aimed to assess the cross-sectional relationships between compulsive exercise, QoL, psychological distress (anxiety and depressive symptoms, and obsessive-compulsive traits) and motivation to change in patients with AN.

**Method:**

A total of 78 adults with AN participated in this study, which was nested within a randomized controlled trial of psychological treatments for AN. At baseline (pre-treatment), participants completed questionnaires assessing compulsive exercise, eating disorder (ED) psychopathology, QoL, psychological distress and motivation to change.

**Results:**

Baseline correlational analyses demonstrated a moderate positive relationship between compulsive exercise and ED psychopathology, and a weak positive relationship between compulsive exercise and psychological distress. There was a moderate negative relationship between compulsive exercise and eating disorder QoL.

**Conclusions:**

These results indicate compulsive exercise is moderately associated with poorer QoL and weakly associated with higher distress. Targeting compulsive exercise in the treatment of anorexia nervosa may help reduce the burden of illness and improve patients’ engagement in treatment.

**Trial registration:**

ACTRN12610000585022. Taking a LEAP forward in the treatment of anorexia nervosa: a randomized controlled trial. NHMRC grant: 634922.

## Plain English summary

Many people with anorexia nervosa (AN) engage in compulsive exercise as a part of their eating disorder. This type of exercise can be defined as very driven and rigid exercise behaviours and attitudes. Although compulsive exercise has been linked to poorer physical and mental health outcomes, little is known about how it impacts on an individual’s quality of life, and their motivation to change their eating disorder. We conducted a study with 78 adults with AN (including 4 males) who were taking part in a larger eating disorder treatment study. Before they started treatment, participants completed questionnaires which asked about eating disorder symptoms, compulsive exercise, quality of life, symptoms of anxiety, depression and obsessive-compulsive features, and their motivation to change. Our results found that people who exercised compulsively experienced more severe eating disorder symptoms. They also demonstrated poorer quality of life relating to their eating disorder and more elevated symptoms of depression and anxiety. Focusing on compulsive exercise in treatment for AN may help patients to improve their quality of life and mood.

## Background

Anorexia nervosa (AN) is associated with a variety of negative physical, psychological and psychosocial effects [1], as well as functional impairment and reduced quality of life (QOL) [2, 3]. Adding to the illness burden are common co-morbidities such as obsessive-compulsive disorder (OCD) and/or obsessive-compulsive traits as well as low levels of treatment engagement [4, 5]. An important aspect of AN psychopathology, given scarce attention in the QoL and treatment engagement literature, is compulsive exercise. This is lacking despite the high prevalence of problematic exercise, its association with compulsivity, and detrimental effects on treatment and illness course [6–8].

Compulsive exercise can be described as an individual’s extremely driven and inflexible exercise patterns, together with a perceived lack of capacity to stop exercising, despite awareness of its negative effects [9]. It has been associated with higher levels of psychological distress in AN, including greater depressive and anxious traits. A conceptual model of compulsive exercise [10] emphasizes both the positively and negatively reinforcing contributions of this type of exercise on affect regulation. Although patients exercise to improve their mood, compulsive exercise can also be maintained by the individual to reduce high levels of negative emotions, primarily anxiety [11, 12]. It is often performed to avoid withdrawal symptoms such as irritability and low mood. These symptoms can occur when an individual is unable to exercise due to treatment restrictions/recommendations [13, 14].

In community samples, two characteristics of exercise have been significantly associated with lower QoL and higher ED psychopathology: (1) feeling guilty when unable to exercise; and (2) exercising predominantly to change weight or shape [15–17]. These findings in the community underline the need to explore associations between compulsive exercise, QoL and ED psychopathology in patients with AN.

Another important facet of AN which can impact upon treatment is the presence of obsessive-compulsive traits. These are a common comorbidity of AN [18] and are closely related to the concept of compulsivity, a key maintaining factor in Meyer et al.’s cognitive-behavioural model of exercise [10]. A positive association between problematic exercise in AN and obsessive-compulsive personality disorder (OCPD) traits has been demonstrated [19]. In contrast, studies of the relationship between obsessive-compulsive disorder (OCD) and excessive exercise have been inconsistent [19]. It may be that examining obsessive-compulsive traits could be more relevant in patients with AN. An updated conceptualization of compulsive exercise and a corresponding self-report measure have since been developed [9] and this relationship can now be examined more closely.

Low motivation to change can also have a significant impact upon progress in treatment for AN, and has been associated with lower BMI [20], slower weight gain [21] and poorer overall QoL [22]. However, little is known about the relationship between compulsive exercise and motivation to change. As compulsive exercise has been associated with other indicators of eating disorder severity such as being a predictor of relapse [23] and having longer hospitalizations [24], it would be interesting to assess whether there is an association between exercise and motivation to change before treatment. Many patients with AN endorse compulsive exercise driven by shape and weight concerns, and as a method of affect regulation [10]. This is an area we wish to explore as it can be argued that these characteristics could contribute to reduced motivation to change. The trans-theoretical model of motivation to change [25] has been applied to patients with AN and posits that patients (with various illness diagnoses) can move between 6 stages of change ranging from pre-contemplation to maintenance and termination.

In summary, compulsive exercise is a core feature of AN, which can add great complexity to the course of the illness and have a negative impact upon treatment [6, 12]. Furthermore, exercise is associated with greater eating disorder psychopathology and higher levels of depression and anxiety. The relationship between compulsive exercise and QoL has been demonstrated in non-clinical studies but needs to be replicated in the clinical population. Little is known about the relationships between compulsive exercise and QOL, and between compulsive exercise and motivation to change in patients with AN. As obsessive-compulsive traits can compromise treatment outcome, the relationship between exercise in AN and these traits also requires further examination.

The current study thus aimed to assess the relationships between severity of compulsive exercise with subjective ratings of eating disorder psychopathology, QOL, psychological distress and motivation to change pre-treatment. It was hypothesized that compulsive exercise would be negatively related to QOL, but positively related to psychological distress. Given the paucity of previous research investigating compulsive exercise and motivation to change, no specific hypothesis was made for this relationship.

## Method

### Participants

A total of 78 adults (including 4 males) participated in the current study, all of whom were enrolled in a multi-site randomized controlled trial (RCT) “Taking a LEAP forward in the treatment of anorexia nervosa” [26]. The trial took place in Leicester, United Kingdom; Sydney, Australia; and New York, United States of America. The main aim of the parent trial was to examine a novel cognitive-behavioural therapy module for compulsive exercise (compu**L**sive **E**xercise **A**ctivity thera**P**y or LEAP) [27], and to determine if it improved treatment outcomes for patients with AN who exercise. Participants were randomly allocated into one of two groups. The first group completed 34 sessions of mCBT-AN [28], a cognitive-behavioural therapy (CBT) which aims to normalize eating and help patients reach and maintain a healthy weight. The second group had eight sessions of LEAP treatment, and 26 sessions of mCBT-AN. LEAP aims to provide psychoeducation to patients about distinctions between healthy/balanced and compulsive exercise, utilizing CBT techniques to manage unhelpful exercise behaviours and beliefs [27]. A pilot study of LEAP for inpatients with AN has demonstrated its acceptability [29].

All participants were recruited for the trial through referral from an eating disorder clinic/service, or from public advertising. Potential participants had to be 18 years or older to enrol in the trial, have a DSM-5 primary diagnosis of AN [30] using the Eating Disorder Examination (EDE) interview [31], and have a Body Mass Index (BMI) between 14 and 18.5. Participants were assessed for medical stability and this was monitored regularly throughout the trial. To enrol in the trial, participants had to have participated in exercise in the previous month, as indicated on the Exercise Participation Screening Questionnaire (participated in an activity on at least one occasion in the previous 4 weeks) or scored at least one time in the past month on the Eating Disorder Examination Questionnaire, i.e. self-report that he/she participated in at least one occasion of exercise in the past 4 weeks (EDE-Q) [32]. This criterion was broad as the study aimed to recruit participants performing a range of exercise behaviour, but participants did not need to view their exercise as compulsive. Exclusion criteria were a diagnosis of psychosis or bipolar disorder, current substance dependence, high suicidality, medical instability and concurrent treatment for their eating disorder. There were 574 potential participants who expressed interest in the trial. However, 496 were deemed ineligible or did not wish to participate, hence there were 78 who were enrolled and randomized in the trial.

Ethics was approved at each site: the Western Sydney University Human Research Ethics Committee; National Health Service Research Ethics Committee in the UK, as part of the Health Research Authority; and the Institutional Review Board at Columbia University in New York, USA. Participants gave written informed consent for the treatment trial and for completion of research assessments.

The mean age of the participants at initial assessment in the current study was 27.38 years (SD: 9.22, median: 24.7, interquartile range: 20.1–31.0 years), their mean BMI was 16.52 (SD: 1.12, median: 16.8, interquartile range: 15.7–17.5) and the average duration of illness (from diagnosis of AN to enrolment in the trial) was 5.65 years (SD: 7.88, median:1.40; interquartile range: .6–8.5 years). 71.8% met criteria for AN-Restrictive subtype, whilst 28.2% met criteria for AN Binge-eating/Purging subtype. Of the participants, 32.1% were born in Australia, 50% were born in the UK, 11.5% in the USA, and the remaining 6.4% were born elsewhere. A total of 52.5% of the sample had completed university, whilst a further 35.9% had completed at least 10 years of schooling. At the time of the study, 57.7% worked full-time or part-time, 26.9% were students, 12.8% were unemployed and 2.6% completed full-time house duties. Most participants were single (69.3%), 26.9% were married or living in long-term relationships, and 3.8% were separated or divorced.

### Measures

Participants completed self-report questionnaires assessing ED psychopathology, compulsive exercise, psychological distress, motivation to change and quality of life. These questionnaires were presented to participants in the order below.

*Eating Disorder Examination-Questionnaire* (EDE-Q) [32] is a 36-item questionnaire, comprised of the four subscales in the EDE interview [31]. It has demonstrated good psychometrics in clinical samples [33]. Participants completed the full EDE-Q, but only the EDE-Q global score was used in the analyses. Higher scores indicate greater eating disorder psychopathology. The Cronbach’s α for the EDE-Q global score in the current study was .94.

*Short Form-12 Health Status Questionnaire* (SF-12) [34] assesses quality of life by investigating the functional limitations related to physical and mental health conditions. It consists of 12 Likert-style response items that examine a range of domains including Physical Functioning e.g. “*Does your health now limit you in these activities? Moderate activities, such as moving a table, pushing a vacuum cleaner, bowling or playing golf”;* Bodily Pain e.g. “*During the past four weeks*, *how much did pain interfere with your normal work, including both work outside the home and housework?”;* and Social functioning e.g. *“During the past four weeks, how much of the time has your physical health or emotional problems interfered with your social activities like visiting friends or relatives?”* This study used two subscales, the Physical Health Component Summary Scale (PCS), and the Mental Health Component Summary Scale (MCS). Higher scores indicate a greater level of functioning. The SF-12 has previously demonstrated good psychometric properties [34] and the Cronbach’s α for the overall scale was .89. The SF-12 can compare QoL across a variety of illness categories [35, 36].

*Kessler-10 item distress scale* (K-10) [37] is a measure of psychological distress (operationalized as anxiety and depression symptoms), originally developed as a screening tool for these symptoms in community samples. The 10-item questionnaire includes questions such as *“Over the past four weeks (28 days), how often have you felt so nervous that nothing could calm you down?”* and “*Over the past four weeks (28 days), how often have you felt that everything was an effort?”.* Higher scores (maximum score of 50) indicate greater psychological distress, with large community surveys by the Australian Bureau of Statistics classifying scores of 30–50 to be in the Very High distress range [38]. It is a valid measure for patients with eating disorders [39]. The total score was used in the current study, and the Cronbach’s α was .92.

*Padua Inventory* (Padua) [40] is a 39-item measure of psychological distress, specifically assessing obsessive-compulsive traits. It consists of six subscales: Contamination obsessions and washing compulsions e.g. *“I feel my hands are dirty when I touch money”;* Dressing/Grooming compulsions e.g. *“I feel obliged to follow a particular order in dressing, undressing, and washing myself”*; Checking compulsions e.g. *“I have to do things several times before I think they are properly done”;* Obsessional thoughts of harm to self/others e.g. *“I think or worry at length about having hurt someone without knowing it”;* and Obsessional impulses to harm self/others e.g. *“When I see a train approaching, I sometimes think I could throw myself under its wheels”.* It has demonstrated good psychometric properties [41] and is valid for use in clinical samples. Higher scores indicate greater obsessive-compulsive traits. The mean total score was used in the current study and the Cronbach’s α for the scale was .93.

*Eating Disorder Quality of Life* (EDQoL) [42] is a 25-item measure of eating disorders-specific quality of life with four subscales: Psychological *“How often has your eating/weight made you feel worse about yourself?”*; Physical/Cognitive “*How often has your eating/weight affected your ability to pay attention when you wanted to?”*; Work/School “*How often has your eating/weight led to a leave of absence from work?”*; and Financial “*How often has your eating/weight led to the need to spend money from savings or use your credit card frequently?”.* These are summed for a mean total score, with higher scores representing lower quality of life. The EDQoL has been previously used in patients with eating disorders [36] and patients with chronic AN [35]. The Cronbach’s α for the overall scale was .93. ED-specific QOL measures can be used to compare results of different treatment interventions [35, 36].

*The Anorexia Nervosa Stages of Change Questionnaire* (ANSOCQ) [43] is a validated measure of motivation to change in patients with AN. It consists of 20 items, assessing three factors of Weight Gain (readiness to achieve a minimum healthy body weight), Eating, shape and weight concerns (readiness to change shape and weight importance in defining achievement and satisfaction in life), and Ego-alien aspects (readiness to change interpersonal issues associated with AN). Each item has 5 answers representing the different stages of change: pre-contemplation; contemplation; preparation; action; and maintenance. The participant marks the response/s that is most in line with their current attitudes. Example items include Weight gain (pre-contemplation response) *“As far as I am concerned I do not need to gain weight”;* Eating, shape and weight concerns (action response) “*I often try to challenge the importance that I place on my body shape or weight in determining my happiness and success”;* and Ego-alien aspects (maintenance response) “*The problems in my relationships with others have improved and I am trying to keep it this way”.* Higher scores indicate greater motivation to change. The following mean scores correspond to the different stages of change: < 1.5 = pre-contemplation; 1.5–2.4 = contemplation; 2.5–3.4 = preparation; 3.5–4.4 = action; > 4.5 = maintenance. Cronbach’s α for the scale was .91.

*Compulsive Exercise Test* (CET) [9] is a 24-item self-report questionnaire examining the core maintaining factors of compulsive exercise for patients with eating disorders. It consists of 5 subscales: Avoidance and rule-driven behavior (CET-Avoidance, e.g. “*I usually continue to exercise despite injury or illness, unless I am very ill or too injured”*); Weight control exercise (CET-Weight control, e.g. “*If I feel I have eaten too much, I will do more exercise*”); Mood improvement (CET-Mood, e.g. *“I feel less stressed and/or tense after I exercise”);* Lack of exercise enjoyment (CET-Lack of enjoyment, e.g. “*I find exercise a chore”*) and Exercise rigidity (CET-Rigidity, e.g. *“I like my days to be organized and structured, of which exercise is just one part”).* The CET-Total score is used in this study, calculated by summing the means for the five subscales. Higher scores reflect more compulsive exercise. It is psychometrically sound and has been validated for use in adult clinical samples [44, 45]. A clinical cut-off score of 15 is effective in differentiating between compulsive and non-compulsive exercise in patients with diagnosed eating disorders [44]. In the current study, only the CET-Total was used, and its Cronbach’s α was .92.

### Data analysis

The Shapiro-Wilk test demonstrated that the data were not normally distributed for the EDE-Q, Padua Inventory, ANSOCQ and CET, thus non-parametric tests (Spearman’s rho) were used in the analyses. Due to hypotheses, one-tailed tests were used, except for the ANSOCQ which used a two-tailed test. The significance level was set at *.*05 and analyses were performed using SPSS, Version 22.

## Results

### Correlations between compulsive exercise, QOL, distress and motivation to change

The aim of this study was to examine the relationships between compulsive exercise, QOL, psychological distress and motivation to change prior to treatment (see Table 1). CET-Total was positively and moderately associated with higher eating disorder psychopathology (EDE-Q). CET-Total was not significantly associated with the SF-12 Mental health Component score, but it showed a weak negative association with the SF-12 Physical health Component Score, meaning poorer physical health quality of life. CET-Total was moderately associated with higher EDQOL scores, indicating poorer quality of life.

**Table 1:** Spearman’s correlations between compulsive exercise and variables of interest at baseline

Measure (*N* = 78)	M	SD	CET-Total (M = 16.26, SD = 4.39)	*p*
Eating Disorder Examination-Questionnaire (EDE-Q)	3.99	1.40	.41**	<.001
SF-12 Physical health Component Score (SF-12 PCS)	46.74	10.43	−.21*	.03
SF-12 Mental health Component Score (SF-12 MCS)	29.32	11.83	−.18	.05
Kessler-10 (K-10)	31.04	9.54	.23*	.02
Padua Inventory (Padua)	23.68	19.52	.23*	.02
Eating Disorder Quality of Life (EDQOL)	1.69	.68	.36**	<.01
Anorexia Nervosa Stages of Change Questionnaire (ANSOCQ)	2.61	.63	−.22	.05

* = *p* < .05, ** = *p* < .01

Our results demonstrated a weak positive association between CET-Total and psychological distress, as measured by both the Kessler-10 and the Padua Inventory. There was no significant association between CET-total and motivation to change (ANSOCQ overall stage score).

## Discussion

The current study aimed to assess the relationships of compulsive exercise to QOL, psychological distress and motivation to change pre-treatment. Confirming the hypotheses, baseline correlations demonstrated weak positive associations between compulsive exercise (CET-Total) and psychological distress (anxiety and mood symptoms on the K-10), aligning with previous research [11, 46]. There was also a weak positive relationship between exercise and obsessive-compulsive traits, confirming previous results [8, 47, 48]. However, this contrasted Bewell-Weiss and Carter’s study [49], which also used the Padua Inventory and found excessive exercise to be negatively associated with obsessive-compulsive symptomatology. Their study utilized a quantitative definition of compulsive exercise and categorized patients as excessive exercisers if they endorsed obligatory exercise for at least 1 h per day, on at least 6 days per week for 1 month. Our use of the CET examines the cognitive-behavioural characteristics of compulsive exercise, rather than the obligatory and quantitative aspects of compulsive exercise which may account for our different results.

A novel aspect of the current study was the examination of the relationships between compulsive exercise and quality of life. At baseline, compulsive exercise was moderately associated with poorer ED-related quality of life (EDQoL) and weakly associated with lower general physical-health quality of life (SF-12 PCS). There are few studies reporting upon these relationships in a clinical sample of outpatients, and our results emphasize the detrimental impact exercise can have upon patients’ general functioning. Mond and Calogero [50] found that the two factors which differentiated between eating disorder patients and healthy women were the same as those associated with poorer quality of life in community samples, i.e. exercising for shape and weight reasons, and avoidance of guilt if exercise was missed [15–17]. However, they did not specifically investigate quality of life in this study. Other research examining an Australian sample of inpatients with EDs found a significant positive relationship between QOL and driven exercise [51]. Their study used the Quality of Life-Eating Disorders measure (QOL-ED) from the computer-generated Eating and Exercise Examination (EEE-C) [52] but looked only at the frequency of exercise and did not directly assess the compulsive qualities of exercise.

As compulsive exercise is associated with a wide range of physical health complaints [8], it was expected that there would be a significant relationship between compulsive exercise and poorer physical health QoL (measured by the PCS on the SF-12) prior to treatment. However, an unexpected finding was that the association between compulsive exercise and the mental-health component of the SF-12 (MCS) approached significance but was not significant (*p* = .05). The MCS measures the functional impairment from emotional issues upon work and social activities. Patients may experience their exercise to be adaptive, in that the behavior serves as both a way to improve their mood and to avoid/manage feelings of guilt and depression if they are unable to exercise. This could have impacted upon their ratings of functional impairment. Although the SF-12 has been evaluated as a sensitive measure of changes in ED pathology [35], it may also be that the MCS is not as sensitive to the positively and negatively reinforcing functions of compulsive exercise behavior.

Compulsive exercise was not significantly associated with lower motivation to change (ANSOCQ) in this pre-treatment sample (*p* = .055). However, we know that patients with a lower level of motivation for change demonstrate a greater rigidity of ED beliefs and are more ambivalent about reducing their ED behaviours. Given these negative characteristics of the illness are associated with poorer motivation to change, it is integral that therapists and medical specialists accurately assess patients’ motivation to change. Future research should investigate this relationship longitudinally across treatment, as it may be that focusing on and addressing exercise in treatment [53] is more important for those who demonstrate lower motivation to change.

There were several strengths in the current study, namely that it incorporated both ED specific and general health QOL measures, as this has been recommended in recent reviews in the field [35, 36]. This study is novel in that no previous research has specifically investigated the relationships between compulsive exercise, quality of life and motivation to change in a clinical sample of outpatients with AN. It did include a small group of male patients with AN, but unfortunately it was not possible to conduct analyses based on gender due to our sample size. Men may experience more functional impairment than women from some ED symptoms e.g. objective binge eating [54], thus it would be beneficial for future studies to utilize larger samples of males with clinical EDs.

This study utilized a range of quality of life measures, however another eating disorder specific measure of HRQoL, e.g. Clinical Impairment Assessment (CIA) [55] may have been a useful addition. As our participants were medically stable and seen on a regular basis as outpatients, our results may not generalize to inpatients or day program patients. Although most of the correlations between compulsive exercise and variables of interest were significant, some of the correlations were weak in magnitude, e.g. K-10, Padua and SF-12 PCS. Finally, our patients were enrolled in an RCT addressing compulsive exercise and it was a criterion of entry that they had participated in at least one form of physical activity in the past month. These low exercise inclusion criteria were used to generalize as best as possible to patients with AN who engage in exercise (of any frequency), however it is important to acknowledge that these results may not generalize to all patients with AN. Despite having a low entry threshold for exercise, the mean CET score in this sample still fell above the clinical cut off score of 15 indicated for patients with AN who are compulsive exercisers [44].

## Conclusions

This study demonstrated that greater compulsive exercise is moderately associated with poorer ED quality of life, and higher levels of ED psychopathology. Greater compulsive exercise is also weakly associated with higher levels of psychological distress. Addressing compulsive exercise is integral to reducing the burden of illness from AN and may be important in improving people’s engagement in treatment. Further research is required to investigate the relationship between these variables, and to utilize longitudinal designs to assess if severity of compulsive exercise predicts these factors over time and throughout treatment.

## Abbreviations

AN: Anorexia Nervosa
ANSOCQ: Anorexia Nervosa Stages of Change Questionnaire
BMI: Body Mass Index
CBT: Cognitive-Behavioural Therapy
CE: Compulsive Exercise
CET: Compulsive Exercise Test
DSM-5: Diagnostic and Statistical Manual of Mental Disorders 5th edition
ED: Eating Disorder
EDE-Q: Eating Disorder Examination-Questionnaire
EDQoL: Eating Disorder Quality of Life scale
K-10: Kessler 10-item Distress Scale
LEAP: Loughborough Eating disorders Activity theraPy
MCS: Mental Health Component Summary Scale
OCD: Obsessive-Compulsive Disorder
OCPD: Obsessive-Compulsive Personality Disorder
PCS: Physical Health Component Summary Scale
PI: Padua Inventory
QOL: Quality of Life
RCT: Randomized Controlled Trial
SF-12: Short Form-12 Health Status Questionnaire
